# A Structural Biology Approach to Understand Human Lymphatic Filarial Infection

**DOI:** 10.1371/journal.pntd.0002662

**Published:** 2014-02-06

**Authors:** Raghavendra Sashi Krishna Nagampalli, Krishnasamy Gunasekaran, Rangarajan Badri Narayanan, Angela Peters, Rajagopalan Bhaskaran

**Affiliations:** 1 Claflin University, Department of Chemistry, Orangeburg, South Carolina, United States of America; 2 University of Madras, CAS in Crystallography and Biophysics, Chennai, Tamil Nadu, India; 3 Anna University, Centre for Biotechnology, Chennai, Tamil Nadu, India; McGill University, Canada

## Abstract

The presence of aspartic protease inhibitor in filarial parasite *Brugia malayi* (Bm-Aspin) makes it interesting to study because of the fact that the filarial parasite never encounters the host digestive system. Here, the aspartic protease inhibition kinetics of Bm-Aspin and its NMR structural characteristics have been investigated. The overall aim of this study is to explain the inhibition and binding properties of Bm-Aspin from its structural point of view. UV-spectroscopy and multi-dimensional NMR are the experiments that have been performed to understand the kinetic and structural properties of Bm-Aspin respectively. The human aspartic proteases that are considered for this study are pepsin, renin, cathepsin-E and cathepsin-D. The results of this analysis performed with the specific substrate [Phe-Ala-Ala-Phe (4-NO_2_)-Phe-Val-Leu (4-pyridylmethyl) ester] against aspartic proteases suggest that Bm-Aspin inhibits the activities of all four human aspartic proteases. The kinetics studies indicate that Bm-Aspin follows a competitive mode of inhibition for pepsin and cathepsin-E, non-competitive for renin and mixed mode for cathepsin-D. The triple resonance NMR experiments on Bm-Aspin suggested the feasibility of carrying out NMR studies to obtain its solution structure. The NMR titration studies on the interactions of Bm-Aspin with the proteases indicate that it undergoes fast-exchange phenomena among themselves. In addition to this, the chemical shift perturbations for some of the residues of Bm-Aspin observed from ^15^N-HSQC spectra upon the addition of saturated amounts of aspartic proteases suggest the binding between Bm-Aspin and human aspartic proteases. They also provide information on the variations in the intensities and mode of binding between the proteases duly corroborating with the results from the protease inhibition assay method.

## Author Summary

Filariasis is a parasitic infectious tropical disease caused by thread like filarial nematodes. These worms occupy the lymph nodes and in chronic cases they lead to the disease “elephantiasis.” Over 120 million people have already been affected by it, and 40 million are seriously disfigured by this disease. These parasites in human, adopt numerous strategies to hamper the host immune system which can facilitate its survival. The ability of the parasite to modulate the host immune system is a concept which explains the versatility of human filarial parasites. One such interesting concept to understand is the secretion of protease inhibitors by filarial parasites. Recently, an Aspin from parasite *Brugia malayi* was identified and the recombinant protein was biochemically characterized. Aspins get fast gaining importance in the fields like medicine, agriculture and biotechnology. Hence, in this study, the inhibition ability of filarial Aspin against human aspartic proteases is attempted from structural biology point of view. This new knowledge may contribute to a better overall understanding of the mechanism that explains the versatility of human filarial parasite. Since filariasis is more often considered as the disease of poor countries, fight against filariasis is also a fight against poverty.

## Introduction

Lymphatic filariasis is a mosquito borne infection caused by *Wuchereria bancrofti, Brugia malayi or Brugia timori* that affects 120 million people in 73 countries and another 1100 million people are at the risk of contracting this dreadful disease [1], [2]. Infection is initiated when infective mosquito bite the susceptible humans living in the endemic areas. Because of the seriousness associated with this infection, lymphatic filariasis is often considered as the second leading cause of permanent and long-term disability [3].

Though the mass drug administration was initiated as a preventive measure, it had only a limited ability [4], [5]. In addition, the increase in drug resistance has also been observed to most of the drugs in mass drug administration [6], [7]. Since yearly administration of these drugs is required in effective control of infection, there is a risk of raise in resistance against these drugs in parasites. Therefore, there is an immediate need for a multi-thronged approach in controlling this mosquito borne parasitic infection. Combining the structural characterization of the filarial proteins along with the identification of candidate antigens would be an ideal strategy in controlling this infection, especially to achieve the targeted elimination date of 2020, by the Global Programme for Elimination of Lymphatic Filariasis [8].

During the process of infection, all stages of the parasite are constantly exposed to various human proteases. It is interesting to understand how filarial parasites successfully evade or counteract the harmful effects produced by the various human proteases. Under this scenario, several lines of studies suggest that filarial parasites have evolved mechanism to neutralize the harmful effects produced by the human proteases. For example, filarial parasites produce three types of classical protease inhibitors viz., serine protease inhibitors (serpins), cysteine protease inhibitors (cystatins) and aspartic protease inhibitors (aspins) to overcome the harmful effects produced by the human proteases.

The first evidence of protease inhibitors in parasite survival was Taeniaestatin from a non-filarial parasite Taenia *taeniaformis* [9]. Later on, hundreds of protease inhibitors of parasite origin have been identified which possess potential applications in medicine, agriculture and biotechnology [10]. However, the role filarial protease inhibitors was understood only when it was reported that serpins [11] and cystatins [12] were few of the most highly secreted proteins in *Brugia malayi*. The suggested function of serpins and cystatins was to contribute to the longevity of the parasite in the blood stream. Despite the pivotal role of Aspins in host pathogenesis, immune regulation and diagnostic marker [13]–[15], no further characterization has been reported on it.

In this context, an Aspin from *Brugia malayi*, termed as pepsin inhibitor homolog compared to the amino acid composition of PI-3 from *Ascaris Suum* [16], has been characterized as an immunodominant and hypodermal antigen [17]–[18]. In addition to this, we have recently reported the pepsin inhibition activity and physicochemical characterization of Bm-Aspin [19]–[20]. Interestingly, this inhibitor has been reported to inhibit the important human aspartic proteases such as pepsin, renin, cathepsin-E and cathepsin-D. It is worth noting that these aspartic proteases are widely distributed in tissues and are thought to be involved in the regulation of physical activities like, lysosomal biogenesis, protein targeting, antigen processing and presentation by degradation of proteins and peptides [21]–[25].

As these aspartic proteases are believed to play a key role in various physiological activities, understanding the aspartic protease inhibitory mechanism of Bm-Aspin becomes imperative to study its role in parasite survival and its immune evasion strategies. However, lack of Bm-Aspin three dimensional structure limit the ability of understanding aspartic protease inhibition mechanism. In order to unravel the structure-based protease inhibition mechanism, Bm-Aspin's, aspartic protease inhibition kinetics and solution structure determination has been initiated and presented in this study. This work happens to be the first report describing the feasibility of solution structure determination of filarial Aspin and understand its protease inhibitory efficiency from structural biology point of view.

## Materials and Methods

### Human aspartic protease inhibition assay

The aspartic proteases pepsin, renin, cathepsin-E, and cathepsin-D purchased from Sigma, USA and the lab purified Bm-Aspin were used for the assays. Equimolar concentrations of Bm-Aspin and the proteases were mixed and incubated in 100 mM sodium acetate buffer at pH 5.6 for 10 min at 37°C separately. Bm-Aspin initially termed as “pepsin inhibitor homologue” [16] based on the sequence homology with *Ascaris Suum* PI-3 and was believed to follow similar kind of pepsin inhibition. Hence pH 5.6 was considered. The sample conditions and the methods that were chosen to study the protease inhibition by Bm-Aspin were similar to those used by Abu-erreish and Peanasky to study the pepsin inhibition by PI-3 from *Ascaris Suum* [26]. To measure the residual protease activity, 10 µl of the Phe-Ala-Ala-Phe (4-NO_2_)-Phe-Val-Leu (4-pyridylmethyl) ester, the specific substrate for aspartic proteases from a stock of 1 mg/ml, was added [27]. The rate of cleavage of proteases was measured spectrometrically at 310 nm. After incubation at 37°C for 10 min, the mixture was centrifuged for 40 min at 17,091 g. The peptide fragments in the supernatant show absorption at 310 nm. The absorption is proportional to the digestion of substrate by the proteases. The whole experiment was repeated six times and the mean values obtained are graphically represented.

The kinetics of aspartic protease inhibition by Bm-Aspin was determined by the UV- spectroscopic method [28]. Using a fixed quantity of the human proteases (5 mM) and fixed reaction time, the rate of proteolysis in the presence of inhibitor was measured. Human aspartic proteases and the proteases pre incubated with increasing concentration of Bm-Aspin (1 mM, 2.5 mM and 5 mM respectively) were taken in 100 mM sodium acetate buffer (pH 5.6) for 10 min at 37°C. To measure the residual protease activity, 10 µl of the same substrate from the stock for each aspartic proteases was added. After incubation as before and after the centrifugation, the reaction was monitored by spectrophotometer at 310 nm.

The *K_m_* value for Bm-Aspin was determined by linear regression method from plots of 1/V vs. 1/S, utilizing substrate concentrations of 7–80 µM. Three fixed concentrations of Bm-Aspin as explained above were used to determine the inhibition constant (*K_i_*) against six concentrations of substrate. Assays were carried out in triplicates and the kinetic constants for Bm-Aspin against the human aspartic proteases were determined using Graphpad Prism 2.0 (San Diego, CA).

### Effect of SDS and pH on pepsin activity (Casein Agar plate and UV spectroscopy methods)

In order to show the activity of the protease and its inhibition by Bm-Aspin under NMR conditions, the effects of SDS and pH on the protease were studied. We have performed protease activity assays using Casein Agar plate and also by the UV spectroscopic method. The inhibition by Bm-Aspin has also been looked at these conditions. Fine punch holes were made on Casein agar plate as described by Cheseseman [29]. Five microgram of pepsin and pepsin preincubated with 100 mM SDS for10 min at 37°C was added separately in two different wells. Approximately equimolar Bm-Aspin was added to pepsin and to pepsin-SDS. After the incubation for 10 min at 37°C, these samples were loaded separately into other two different wells. Reaction buffer at pH 5.6, water, 5 mM pepstatin treated pepsin and 5 mM pepstatin treated pepsin-SDS were placed respectively into three different wells as controls. Plates were incubated overnight at room temperature to examine the pepsin activity. To measure the pepsin activity at pH 7.0, similar experimental setup described above was used except that pepsin was dissolved in phosphate buffer at pH 7.0. In UV spectroscopy method, equimolar mixtures of Bm-Aspin with pepsin and pepsin-SDS were incubated separately for 10 min in sodium acetate buffer pH 5.6 and phosphate buffer pH 7.0 at 37°C. To measure the protease activity, the method described above explaining the human aspartic protease inhibition assay was followed. The whole experiment was repeated three times and the mean values obtained are graphically represented

### NMR structural and binding studies

The plasmid containing Bm-Aspin was expressed in ^15^N minimal medium using ^15^NH_4_Cl as the sole nitrogen source. The purification and the refolding protocols are the same that have been described earlier [19]. Each sample for NMR measurement was concentrated to 0.3 mM using an Amicon (MWCO = 10 kDa) ultrafiltration cartridge. The final NMR sample was in the solvent containing 25 mM phosphate buffer (pH 7.0), 100 mM NaCl, 1 mM DTT, 100 mM Sodium Dodecyl Sulfate (SDS) and 10% D_2_O (v/v). This NMR study was initially performed without any detergents and the resultant spectrum suggests the possibility of aggregation. Then the following chemicals/detergents were used to figure out the best one that helps to avoid aggregation: 0.5 M urea and 1% glycerol, 100 mM n-Dodecyl β-D-Maltopyranoside (DDM), 1% *n*-octyl-β-D-glucoside (OG), 1% triton-×100 and 100 mM SDS. The final goal is to obtain the most suitable condition for Bm-Aspin that could provide good quality NMR spectra. There after the protein sample for the NMR experiment was refolded and concentrated in the presence of the above mentioned detergents each time, respectively.

^15^N HSQC spectrum was acquired for Bm-Aspin at 298 K. Data was collected on the Bruker Avance III 700 MHz spectrometer available at Claflin University. The NMR experiments were recorded with the carrier position of 4.678 ppm for ^1^H and 117 ppm for ^15^N. All chemical shifts were referenced to internal D_2_O. The triple resonance NMR experiments like HNCO, HN(CO)CA, HNCA, CBCA(CO)NH and HNCACB on a uniformly ^13^C, ^15^N labeled Bm-Aspin were carried out as mentioned above. The data acquired were processed using NMRPipe [30] and analyzed using Sparky [31]. For understanding the interactions between Bm-Aspin and human aspartic proteases, a series of ^15^N-HSQC spectra were collected by titrating with progressive additions of human aspartic proteases (pepsin, renin, cathepsin-E and cathepsin-D) to ^15^N-labeled Bm-Aspin to attain molar ratios of 0∶1, 0.1∶1, 0.5∶1 and 1∶1 respectively.

## Results

### Human aspartic protease inhibition

**Figure 1:**
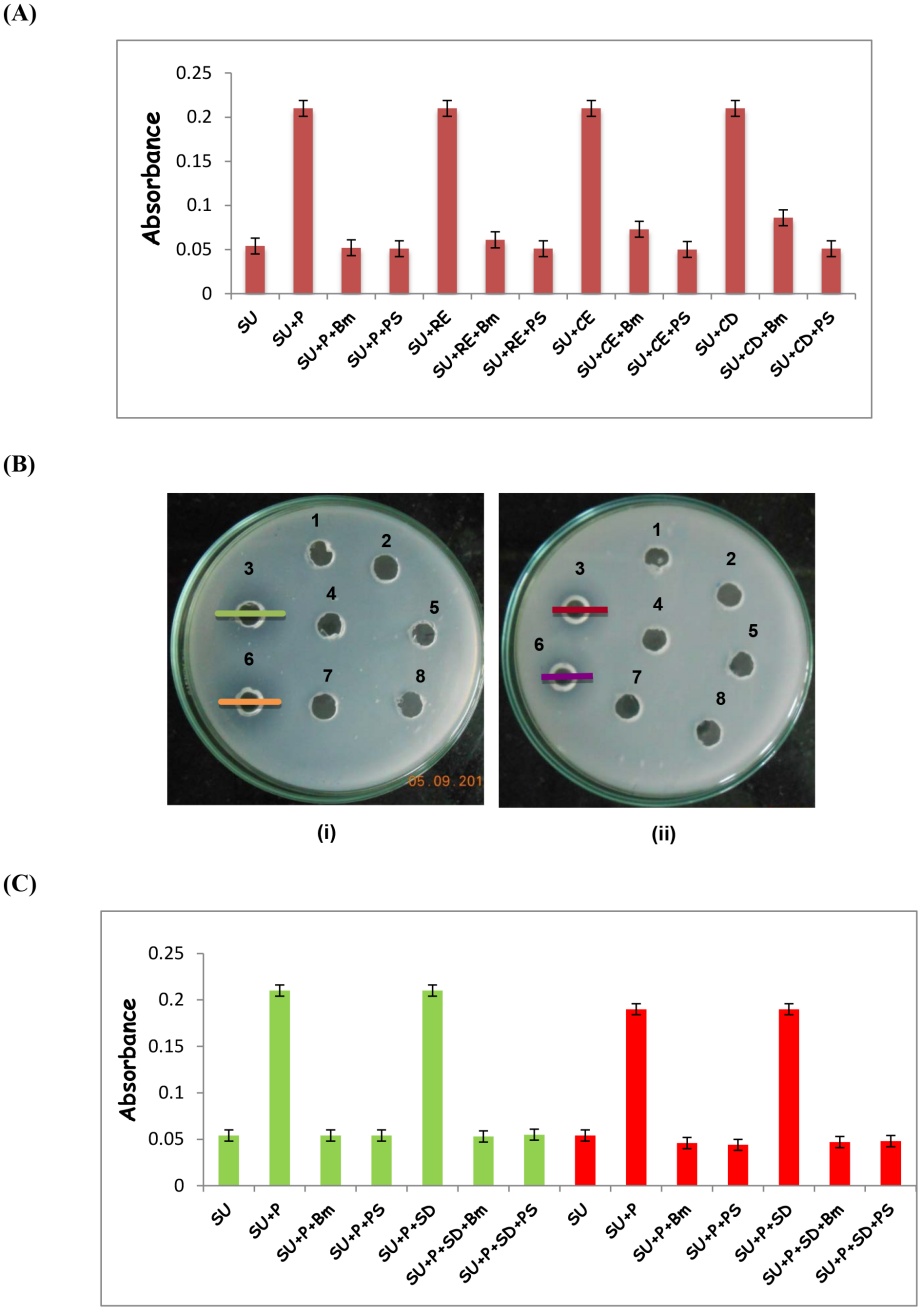
Human aspartic protease inhibition activity. (A) The inhibition activity of Bm-Aspin against human aspartic proteases using UV-spectroscopy; SU: Substrate, P: Pepsin, RE: Renin, CE: Cathepsin-E, CD: Cathepsin-D, PS: Pepstatin, Bm: Bm-Aspin. The values represent the mean of six independent experiments ± SD. (B). The activity of pepsin and inhibition of Bm-Aspin in the presence of 100 mM SDS using Casein agar plate: (**i**) at pH 5.6: Reaction Buffer (100 mM sodium acetate, 10 mM CaCl_2_) (well 1), Water (well 2), 5 µg of pepsin (well 3), Equimolar Bm-Aspin and pepsin preincubated for 10 minutes at 37°C (well 4), 5 µg of pepsin with 5 mM pepstatin (well 5), 5 µg of pepsin preincubated with 100 mM SDS for 10 minutes at 37°C (well 6), Bm-Aspin with SDS treated pepsin (well 7), pepstatin with SDS treated pepsin (well 8) and (**ii**) at pH 7.0: Reaction Buffer (25 mM phosphate buffer, 10 mM CaCl_2_) (well 1), Well 2 to Well 8 contain the same as before. The zone of digestion by pepsin is indicated by a bold dash. (C). The activity of pepsin and inhibition of Bm-Aspin in the presence of 100 mM SDS with pH 5.6 and 7.0 using UV-spectroscopy: SU: substrate, P: pepsin, PS: pepstatin, Bm: Bm-Aspin, SD: SDS. The plots in *green* and *red* represent the activity of pepsin at pH 5.6 and 7.0 respectively. The values represent the mean of three independent experiments ± SD.

Inhibition effect of Bm-Aspin on the human aspartic proteases was examined (Figure 1A). When assayed for the inhibition of proteases by Bm-Aspin using the specific aspartic protease substrate, Phe-Ala-Ala-Phe(4-NO_2_)-Phe-Val-Leu (4-pyridylmethyl) ester, successful inhibitions of all four proteases were observed.

### Effect of SDS and pH on pepsin activity and its inhibition by Bm Aspin

Protease digestion was observed in Casein agar plate by 5 µg of pepsin and 5 µg of pepsin preincubated (for 10 min at 37°C) with 100 mM SDS at both pH 5.6 and 7.0 (Figure 1 B). Encouragingly, no zone of digestion was observed when Bm-Aspin was added to pepsin and pepsin-SDS. In addition to this, pepstatin seems to inhibit both pepsin and pepsin-SDS at both pH 5.6 and 7.0 respectively. The results obtained from the Casein Agar plate method clearly indicate that the protease activity is affected by 9% by the presence of 100 mM SDS and 19% by the raise in the pH to 7.0. UV spectroscopy based analysis also revealed protease activity of pepsin at both the pH 5.6 and 7.0 (Figure 1 C) in the presence of SDS. The results of the UV spectroscopic method indicated that no effect on the protease activity by the presence of SDS and that the pH to 7.0 produced a 10% reduction in the activity. Bm-Aspin was found to inhibit the protease under both the conditions that were used. As expected, pepsin activity was completely inhibited by Bm-Aspin in the presence of SDS both at pH 5.6 and 7.0. From these results, it is clearly evident that pepsin is active at pH 7.0 and in 100 mM SDS. The behavior of the other aspartic proteases used in the study under the above mentioned conditions was believed to be similar. Hence, we felt that it's worth proceeding with the NMR titrations under these conditions to investigate the binding of Bm-Aspin with human aspartic proteases.

### Kinetics of aspartic protease inhibition by Bm-Aspin

**Figure 2:**
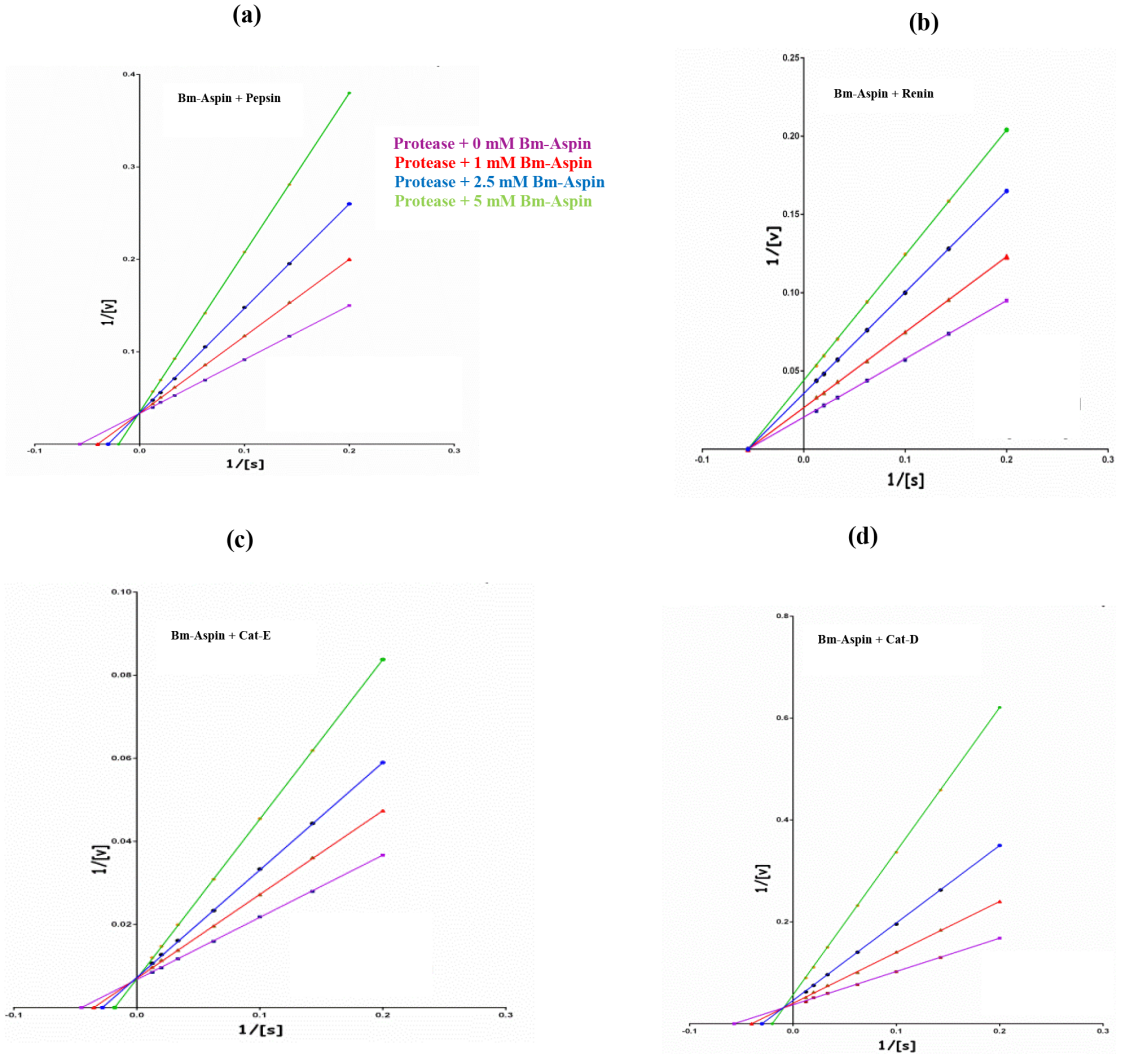
Kinetics of aspartic protease inhibition by Bm-Aspin. Lineweaver-Burk Plots showing the variation (1/V with that of 1/S) of competitive inhibition of pepsin (A) and cathepsin-E (C), non-competitive for renin (B) and mixed inhibition for cathepsin-D (D) respectively. Assays were carried out in triplicates, with the fixed quantity of proteases (5 mM) and varying concentrations of Bm-Aspin (0 mM, 1 mM, 2.5 mM and 5 mM) The inhibition constants were determined using Graphpad Prism 2.0 (San Diego, CA).

The double inverse Lineweaver-Burk plots depicted the inhibition of pepsin, renin, cathepsin-E and cathepsin-D by Bm-Aspin (Figure 2A–D respectively). The rate of the cleavage of the specific substrate was found to be decreased with increasing Bm-Aspin concentration. Inhibition constant (*K_i_*) for pepsin, renin, cathepsin-E and cathepsin-D inhibition by Bm-Aspin was found to be: 2.1 (±0.7) nM, 2.9 (±0.9) nM, 4.3 (±0.2) nM, and 6.5 (±0.6) nM respectively. The data clearly indicate the strength of inhibition among the four different proteases with Bm-Aspin.

### NMR structural studies on Bm-Aspin

**Figure 3:**
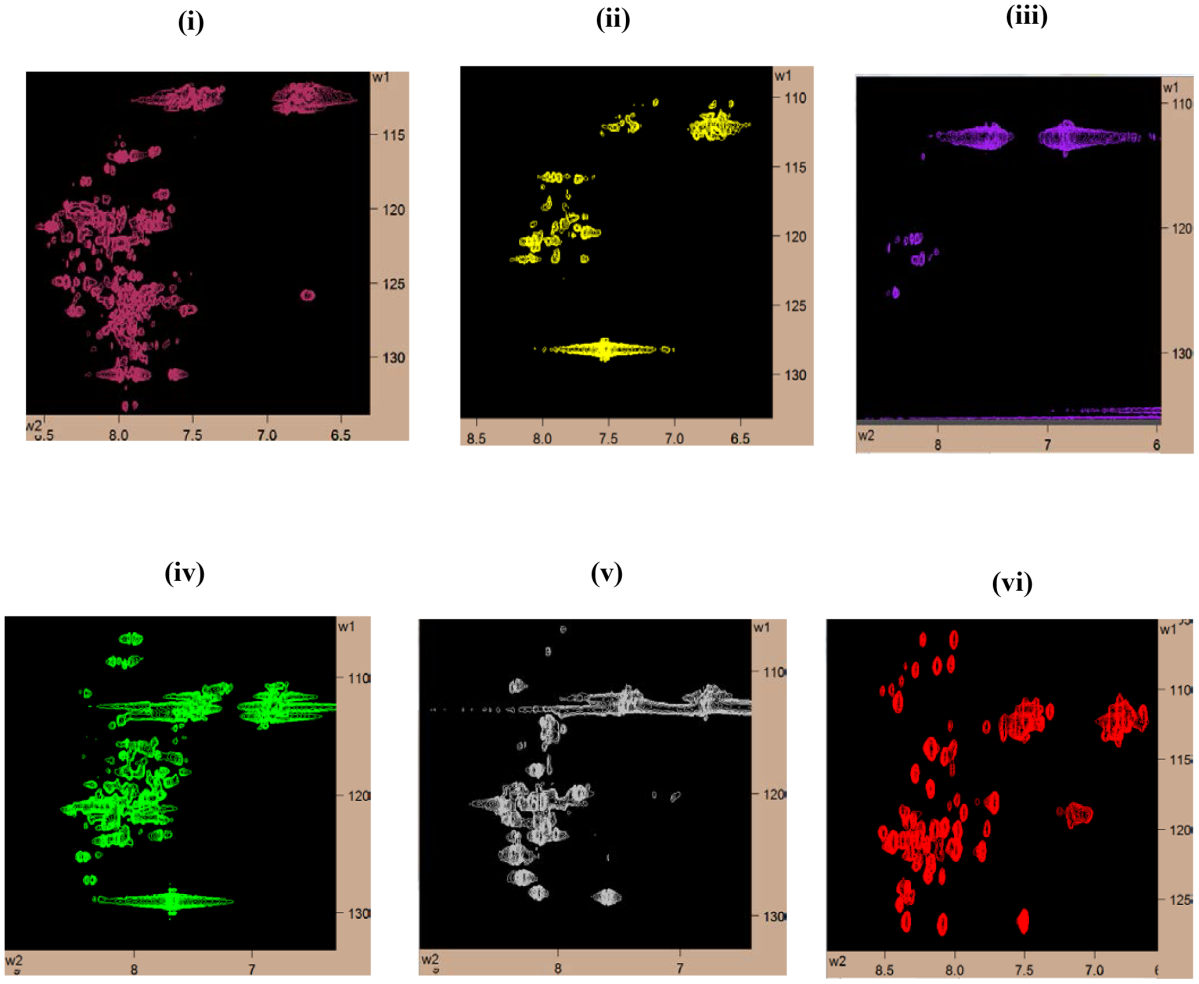
NMR screening on Bm-Aspin with different detergents. ^15^N HSQC spectra of Bm-Aspin at pH 7.0 with the addition of: (i) No detergent (ii) 0.5 M Urea and 1% glycerol (iii) 1% *n*-octyl-β-D-glucoside (OG), (iv) 100 mM n-Dodecyl β-D-Maltopyranoside (DDM) (v) 1% triton X-100, and (vi) 100 mM SDS.

**Figure 4:**
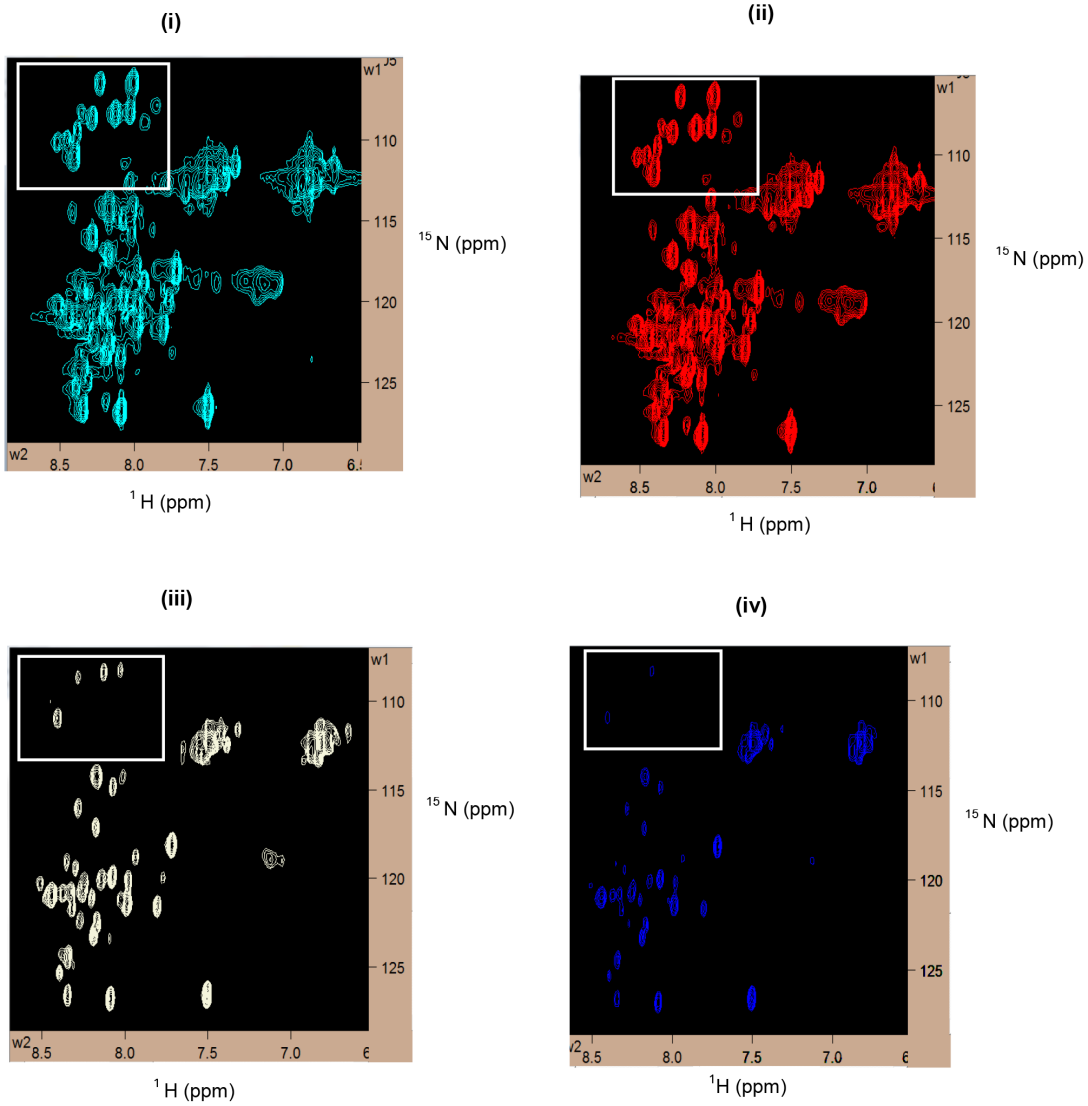
Bm-Aspin ^15^N HSQC spectra at varying concentrations of SDS. Comparison of Bm-Aspin ^15^N HSQC spectra in the presence of varying concentrations of SDS; (**i**) 50 mM SDS, (ii) 100 mM SDS, (iii) 150 mM SDS, and (iv) 200 mM SDS. The inset box indicates the well resolved glycine peaks for comparison to identify the optimum solvent conditions for a well behaved NMR spectrum.

**Figure 5:**
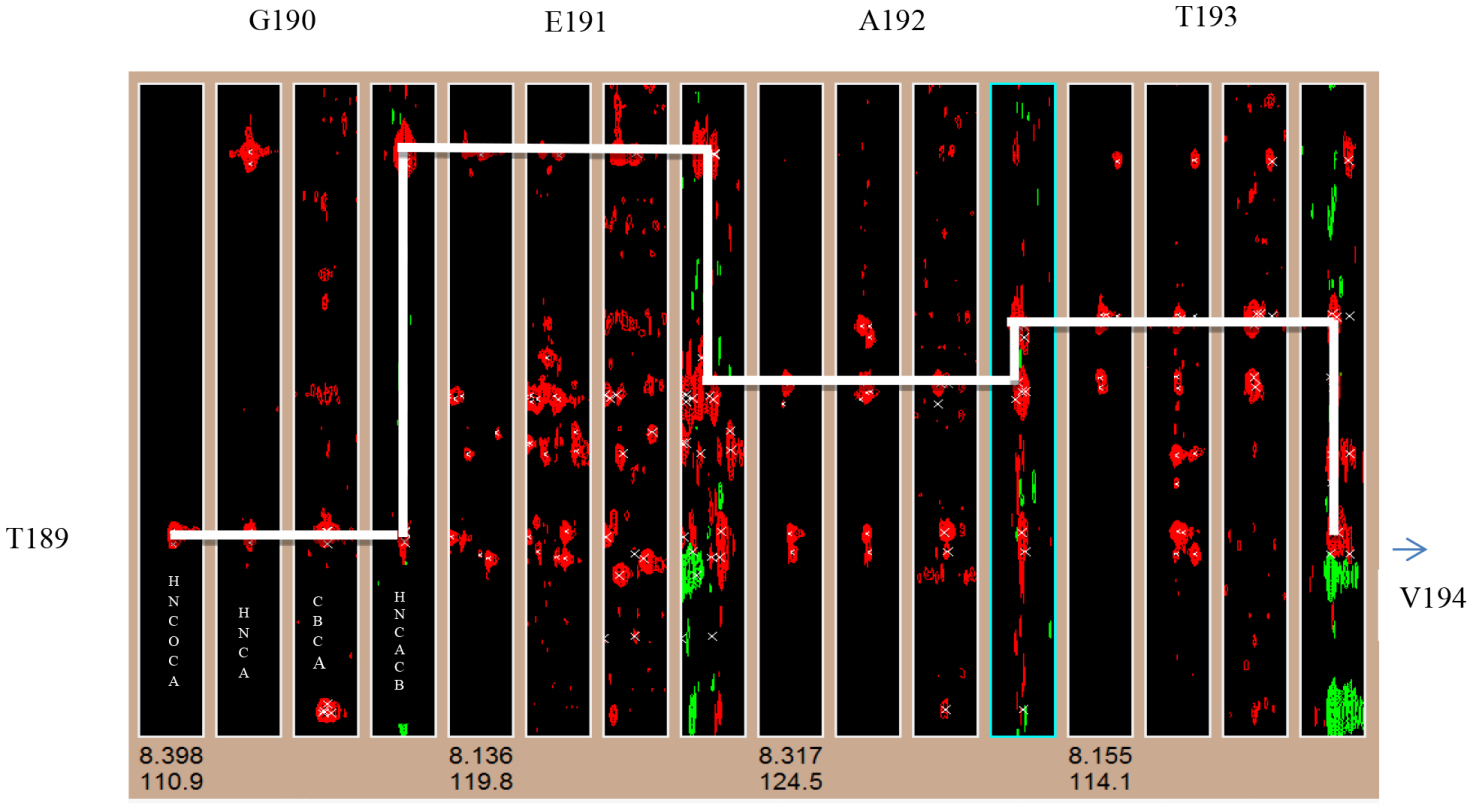
Sequential residue connectivities using triple resonance NMR strip plot. Plot showing the strips of four triple resonance NMR spectra of Bm-Aspin in the following order: HNCOCA, HNCA, CBCACONH and HNCACB. The sequential ^13^Cα connectivities for the residues' stretch T189-V194 are indicated by the continuous line drawn between the adjacent Cα.

The NMR measurements carried out on Bm-Aspin devoid of any detergents resulted in poorly resolved spectrum indicating an aggregated state of the protein (Figure 3(i)). In order to determine the sample conditions for NMR suitability, Bm-Aspin was refolded and screened in the presence of the following five detergents, such as 0.5 M urea, 1% OG, 100 mM DDM, 1% triton X-100 and 100 mM SDS respectively (Figure 3(ii–vi)). The HSQC spectrum is an important qualitative and quantitative tool to validate the uniform folding and structural homogeneity of a purified protein sample. The quality and the number of peaks present in the HSQC spectrum reveal the monomeric or oligomeric nature of the protein. This information is vital to assess the feasibility of further solution NMR based structural characterization. Among the five conditions screened, SDS produced a well resolved spectrum with the dispersion and narrow line widths indicating a well folded non aggregated protein (Figure 3(vi)). The optimum concentration of SDS in the buffer was determined by investigating the effect of SDS on Bm-Aspin by NMR (Figure 4). Our data indicated that the HSQC spectra closely resembled one another when SDS concentration was in the range of 50–100 mM (Figure 4(i–ii)). However, the spectra started to lose their quality at a concentration above 100 mM with missing peaks, indicating that the protein is either getting aggregated and/or partially denatured (Figure 4(iii–iv). Taken together, 100 mM SDS was chosen as the working condition for further characterization (Figure 4(ii). Though the presence of SDS yielded a quality spectrum, observation of lesser number of peaks than expected, that too, with overlap among them was the disappointing factors. The proper folding of the protein was evident when the HSQC peaks corresponding to all the 12 glycines that are evenly distributed throughout the sequence were visible and found well separated (Inset of Fig. 4(ii)). The overlap seen in the middle region of the HSQC spectrum is well resolved in the carbon dimension of the triple resonance spectra. Hence, we have continued to view the carbon dimension, by acquiring triple resonance NMR experiments. These experiments resolve the peak overlaps by separating them on its carbon dimension. The triple resonance experiments for the backbone assignment, like HNCO, HN(CO)CA, HNCA, CACB(CO)NH and HNCACB, were carried out at 298 K. The analysis of 3D HNCO spectra revealed the presence of nearly 95% of the peaks with relatively uniform intensity. The yields of the other triple resonance spectra are in the range of 80 to 90%. Further, well resolved resonances of the complete set of Asn and Gln 21 residue doublets were completely picked up from the triple resonance HNCO spectrum. This indicated that the NMR sample of Bm-Aspin is in a well folded state with a good structural stability and that it is feasible to carry out solution structural studies using NMR. By combining the pairs of 3D NMR spectra for the assignment of Cα and Cα/Cβ, respectively, the backbone assignment of Bm-Aspin is in progress and the complete assignment would soon be published elsewhere. To mention the progress of the backbone assignment process, a strip plot indicating the sequential Cα connectivity for the residue stretch T189 to V194, using the two pairs of triple resonance NMR spectra, namely, HN(CO)CA, HNCA, CBCACONH and HNCACB have been shown in Figure 5.

### Binding studies of Bm-Aspin against human aspartic proteases by NMR

**Figure 6:**
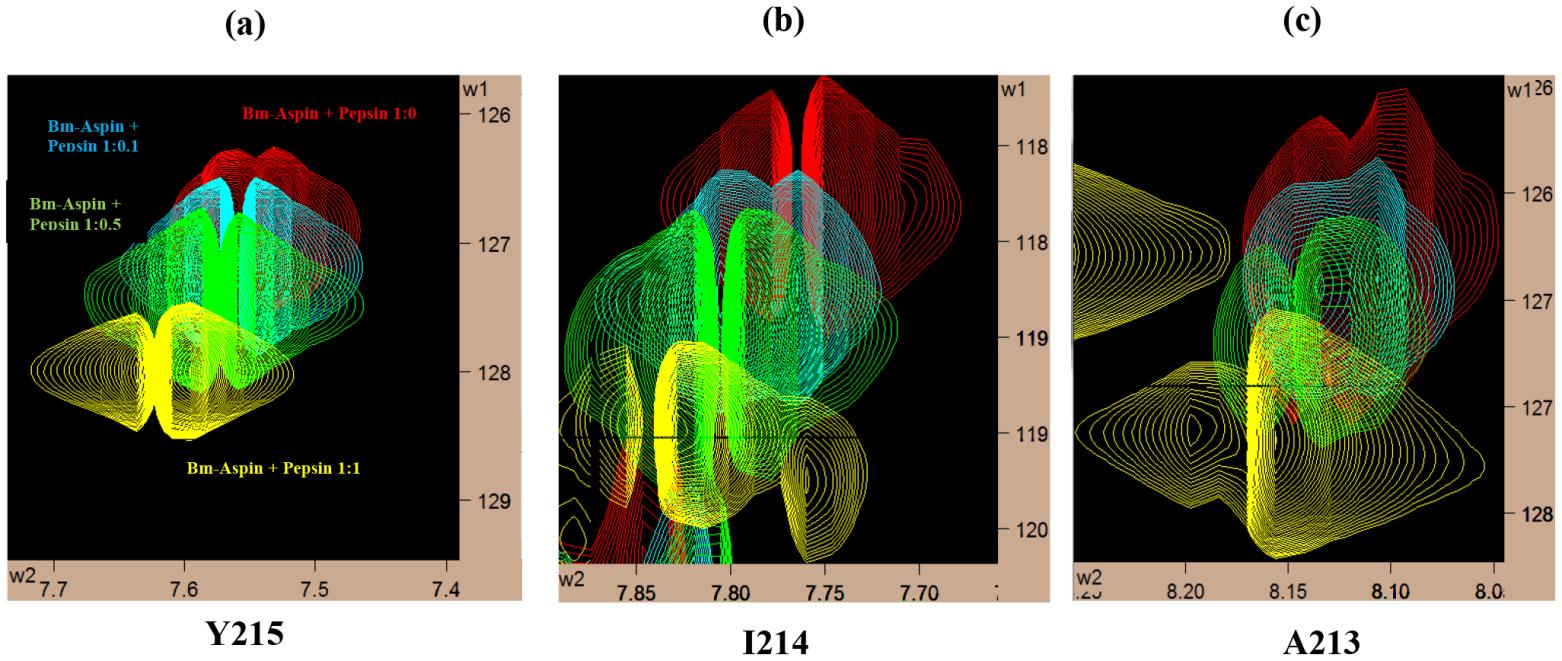
NMR chemical shift perturbations in Bm-Aspin due to pepsin interactions. Perturbations in the chemical shift position of the residues Y215 (a), I214 (b), and A213 (c) respectively in Bm-Aspin upon addition of increasing concentrations of pepsin. Ratios of Bm-Aspin to pepsin are: 1∶0 (red), 1∶0.1 (cyan), 1∶0.5 (green), and 1.1 (yellow).

To determine and analyze the interactions between the Bm-Aspin and the human aspartic proteases, the unlabeled proteases were titrated against the ^15^N labeled Bm-Aspin at four different molar ratios with the last being the saturated one at 1∶1. For each of the titrated sample, the [^1^H ^15^N] HSQC spectrum was acquired. Aspartic protease interactions were followed by monitoring the changes in chemical shift positions in the fingerprint region of Bm-Aspin of the HSQC spectra. The spectra of the set of four titrated samples with a specific protease were overlaid on each other. The residues of Bm-Aspin that have been affected by the protease titrations are seen to be perturbed, shifted away gradually from the reference spectrum that are clearly visible from the superposed spectra. There are nearly 15 common residues that have been affected by the protease interactions, out of which the following residues (G16, G22, G82, G169, G190, A192, A204, A213, I214 and Y215) possess appreciable peak movement indicating their involvement in the interaction phenomena. All the spectra are having the same trend in their protease interaction shifts with pepsin producing more appreciable shifts. The general inhibition characteristics that have been observed from the kinetic and inhibition assay methods are in perfect agreement with the NMR titration method, duly confirming the results. A visual look at the overlaid spectra clearly confirms the strong inhibitory characteristics of pepsin compared to the rest. There is strong evidence about the involvement of the C-terminal residues in the interaction process of all the proteases. Additionally, for pepsin, some glycines from the N-terminal domain are found to be affected as compared to the rest of the proteases, which could attribute to the more inhibitory nature of pepsin. From the binding studies of Bm-Aspin with the aspartic proteases, we have observed that there are five residues (A192, A204, A213, I214 and Y215) of the C-terminal domain that are commonly affected in all the four aspartic proteases interactions. This provides an information that the same kind of interaction mode is followed in all the four protease interactions. Three of the N-terminal domain residues (G16, G22 and G 82) are appreciably affected in interacting with Pepsin and Renin and they are smaller for CatD and CatE. These sites could be the aiding factor for the two former proteases to have higher activities compared to the latter two. These residue specificities for the proteases clearly indicate that they are the genuine interacting sites. As the HSQC spectrum has more peak overlaps, for simplicity, only the residues A213, I214, and Y215 that are having appreciable shifts when interacting with pepsin are shown here as an example. (Figure 6 A–C).

**Figure 7:**
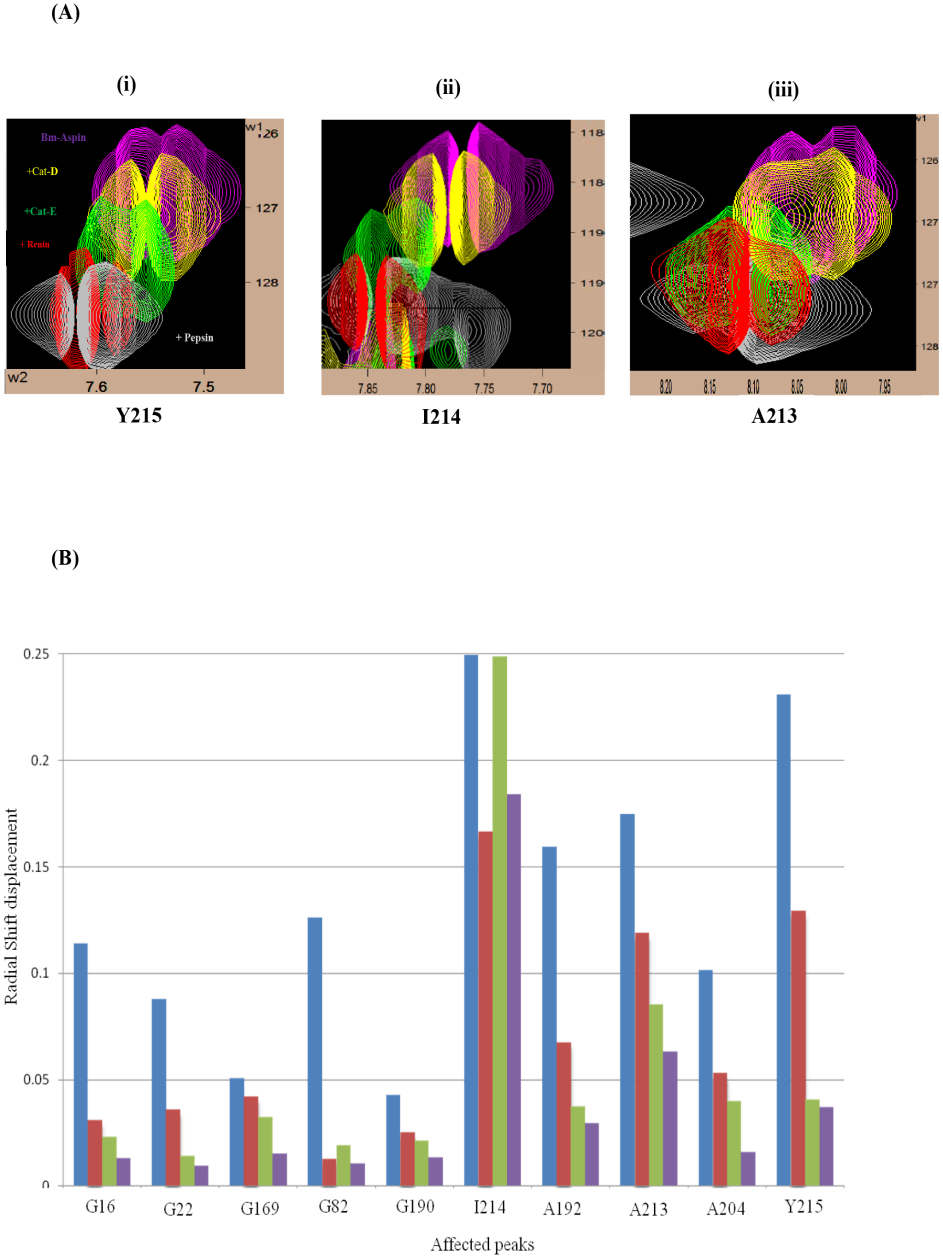
Chemical shift perturbations upon addition of human aspartic proteases. (A) NMR Chemical shift Perturbations in Bm-Aspin due to different protease interactions at their saturated conditions. Chemical shift perturbations of the residues Y215 (i), I214 (ii), and A213 (iii) respectively, upon the addition of human aspartic proteases at their saturation levels. Free Bm-Aspin (Magenta), Bm-Aspin+Cathepsin-D (Yellow), Bm-Aspin+Cathepsin-E (Green), Bm-Aspin+Renin (Red), Bm-Aspin+Pepsin (Grey). (B) Comparison of Bm-Aspin chemical shift perturbation upon addition of human aspartic proteases. The bar diagram indicating the radial shift (calculated for each of the affected residues, by combining both the chemical shifts of ^1^H and ^15^N, using the equation: Radial shift displacement (Δδ) = {(H_f_−H_b_) ^2^+[(N_f_−N_b_)/6] ^2^} ^1/2^. A scaling factor of 6 was used to normalize the differences in the ^1^H and ^15^N spectral widths. H_f_, H_b_, N_f_, and N_b_ are the chemical shifts of each residue's amide ^1^H and ^15^N in the **free** (Bm-Aspin alone) and **bound** (Bm-Aspin+protease complex) states, respectively) in ppm, observed due to NMR chemical shift perturbations in Bm-Aspin with the addition of proteases, (Bm-Aspin+Pepsin in blue, Bm-Aspin+Renin in red, Bm-Aspin+Cathepsin-E in green, Bm-Aspin+Cathepsin-D in magenta), observed for the following 10 residues: G16, G22, G82, G169, G190, A192, A204, A213, I214, and Y215.

On comparing the aspartic protease interactions with Bm-Aspin at their saturated conditions, the saturated sample spectra for each of the four proteases were overlaid on each other. Again, the same three residues considered in the previous example have been shown here (Figure 7A (i–iii)). The peak movements strongly support the earlier observations that Bm-Aspin inhibits pepsin more, followed by renin, cathepsin-E and cathepsin-D in the order of inhibition, respectively. In analyzing and comparing the chemical shift perturbations observed due to the Bm-Aspin interactions with all the four aspartic proteases, the radial shift displacement has been calculated for each of the affected residues, by combining both the chemical shifts of ^1^H and ^15^N, using the following equation:
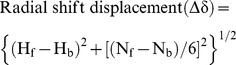
A scaling factor of 6 was used to normalize the differences in the ^1^H and ^15^N spectral widths. H_f_, H_b_, N_f_, and N_b_ are the chemical shifts of each residue's amide ^1^H and ^15^N in the free (Bm-Aspin alone) and bound (Bm-Aspin+protease complex) states, respectively. The bar diagram has been drawn using the radial shifts data obtained for the earlier mentioned 10 residues, when each of the four proteases used and is as shown in Figure 7B. A thorough look of the bar diagram clearly indicates Bm-Aspins' strong binding affinity with pepsin. The C-terminal residues show stronger affinity compared to the N-terminal residues. The molecular modeled structure of Bm-Aspin (not shown), predicted from SWISS-MODEL (swissmodel.expasy.org), has two separate domains, the N-terminal and C-terminal. The present analysis supports the strong interaction of Bm-Aspin C-terminal domain with the proteases. It is this C-terminal domain that has an additional helical secondary structure compared with the crystal structure of *Ascaris Suum inhibitor*, PI-3. Hence, it is quite obvious to expect the role of Bm-Aspin C-terminal domain in the inhibitory actions against the aspartic proteases.

## Discussion

Filarial parasites while gaining entry into the mammalian host are exposed to a variety of hostile factors including human proteases. To circumvent the harmful effects of these proteases, parasites have evolved several counter attacking mechanisms. For example, filarial parasites are known to secrete serpins, cystatins, and aspins to neutralize the human proteases thereby contributing to the longevity of the parasite in the blood stream. Though the functional characterizations of serpins and cystatins have been reported [11], [12], the studies on aspins and its inhibition properties are not fully understood. Our previous studies reported that the *Brugia malayi* Aspin inhibits the important human aspartic proteases [20]. In continuation to our earlier work, the present paper reports the inhibition kinetics using a specific substrate and also reports the feasibility of structure determination of Bm-Aspin by NMR.

Determination of inhibition constant for pepsin inhibition activity of Bm-Aspin was reported with Casein as a substrate in our previous studies [20]. Formation of macroscopic aggregates, hazy zones, and high background with poor readability might sometimes limits the usage of casein as a substrate in monitoring the aspartic protease activity [32]–[34]. In order to overcome this problem, we have used the specific substrate to perform the kinetic studies so that, the results obtained are reliable and highly specific [27]. Before initiating the kinetic studies, the effects of Bm-Aspin on the human aspartic proteases against this specific substrate were examined. An appreciable influence on all the four aspartic proteases was evident with Bm-Aspin. In all cases, addition of equimolar quantity of the Bm-Aspin inhibitor resulted in almost complete inhibition of the proteolytic activity of all the proteases that were examined.

Kinetic studies of Bm-Aspin were performed to find out its mode of protease inhibition. Our data clearly indicates that the protease inhibition by Bm-Aspin was found to be competitive for pepsin and cathepsin-E, non-competitive for renin and mixed for cathepsin-D. Similar trend of linear competitive inhibition was observed with Pepsin when Casein was used as a substrate [20]. Thus, this finding confirms the reproducibility of pepsin inhibition kinetics by Bm-Aspin and also the ability to use Casein as a substrate to determine the inhibition constant.

The mode of pepsin and cathepsin-E inhibition by Bm-Aspin was found to be comparable to that of an Aspin, PI-3 from the non-filarial nematode *Ascaris suum* that infects the pigs [35]. This *Ascaris suum* inhibitor, PI-3, was found to be ineffective against renin and cathepsin-D and in contrast an appreciable influence on all the four aspartic proteases was evident with Bm-Aspin. The role of PI-3 N-terminal end residues was discussed in the case of PI-3 - pepsin crystal structure complex [36]. Based on this observation and sequence similarity of Bm-Aspin with PI-3, (data not shown), N-terminal residues of Bm-Aspin have been hypothesized to play a role in protease inhibition. However, from the NMR chemical shift perturbation analysis discussed above, the role of C-terminal domain has been emphasized to have considerable interactions with all the proteases, with pepsin showing more perturbations. The reason for the inhibition of all the four aspartic proteases by Bm-Aspin may be attributed due to the difference in the nature of N-terminal composition from that of PI3 (data not shown) and the excess of about 66 amino acid residues, (not as a single stretch), present in Bm-Aspin compared to PI-3.

The inhibition constant (*K_i_*) values determined by linear regression of 1/V vs 1/S plot suggests that renin, cathepsin-E and cathpesin-D inhibition by Bm-Aspin are 0.8, 2.2 and 4.4 times less specific than that of pepsin. This correlates with our previous iso-thermal calorimetric titration experiment results indicating that the preference of protease inhibition by Bm-Aspin is in the following order: pepsin>rennin>cathepsin-E>cathepsin-D [20]. Such mode of inhibition was comparable to that of aspartic protease inhibition by pepstatin, suggesting that the protease inhibition of Bm-Aspin is similar to that of pepstatin mode of inhibition [37].

In order to understand the structure based protease inhibition mechanism, solution structural studies of Bm-Aspin has been initiated and in addition to this, the binding of Bm-Aspin against the human aspartic proteases was further confirmed using NMR HSQC titrations as discussed below.

### Feasibility of Bm-Aspin solution structure determination by NMR

Although proteinaceous aspins were proved to be essential and critical in inhibiting the aspartic proteases, very little structural information is known to explain the mode of action. The primary reason may be the difficulty of producing milligram quantities of aspins for structural characterization. Recombinant expression of aspins in *E. coli*, the primary machine for large-scale protein production for structural studies, has had very limited success. As a result, there are only two examples of recombinant expression of an Aspin from prokaryotic sources as of now, for which structural characterization has been reported [36], [38]. We report here the isotopic labeled production of Bm-Aspin from the prokaryotic source which is feasible for structural characterization by solution NMR. This happens to be the first report on attempting solution NMR studies from a protein involved in human lymphatic filariasis.

Many attempts to produce Bm-Aspin suitable for NMR studies without the use of any detergent became unsuccessful. The observation of poorly resolved spectra from each experiment suggested the aggregated state of the protein. From these results, we were convinced that a membrane mimetic environment is necessary during Bm-Aspin refolding and reconstitution. The above assumption can be confirmed based on the evidences from the earlier work suggesting that, Bm-Aspin is a surface protein secreted continuously from *Brugia malayi* at all stages of its life cycle [17]. From this evidence it was hypothesized that Bm-Aspin may contain at least one helix or several surface embedded residues.

As explained above, to carry out the structural determination of Bm-Aspin by solution NMR, detergent screening to find the suitable membrane mimetic environment is an essential prerequisite. The choice of the detergent to be used will be determined by considering the protein solubility, stability and the quality of the ^15^N HSQC NMR spectrum. The HSQC spectrum correlates the amide proton and the corresponding nitrogen pair of each residue within a protein and provides a map of the finger print region. It also serves as a building block for multidimensional NMR experiments upon which the resonance assignments and the determination of the 3D structure of a protein rely. Thus, obtaining a well resolved HSQC spectrum is important for structural characterization by solution NMR.

Five conditions were screened to obtain the suitable sample of Bm-Aspin to carry out solution NMR studies. These include the combination of urea and glycerol [39], which has been successfully proved in some cases to prevent aggregation after refolding and OG, the common detergents for membrane protein crystallization [40], SDS and triton X-100, which are commonly used for solution NMR [41], [42]. For most of the detergents that were used, the protein appeared to be in aggregated state leading to poorly resolved spectra with broader line widths. In contrast, SDS yielded a good quality HSQC spectrum with more number of well resolved peaks and limited number of peak overlaps in comparison to the rest of the detergents that were screened. In fact, SDS has served as one of the most popular membrane mimetic that has been widely used for integral membrane protein structure and function studies [43], [44]. Glycines have a specific nitrogen chemical shift range and there are 12 Glycine residues that are distributed evenly throughout the sequence of Bm-Aspin. The presence of peaks around 106–110 ppm of the nitrogen chemical shift region in the HSQC spectrum have been assigned to all the glycines. This gives a clear indication that the protein is well folded and that it is suitable for the NMR solution structure determination and other structural studies. Though SDS yielded a good HSQC spectrum of Bm-Aspin, a severe overlap of the peaks in the proton chemical shift region around 8 ppm, was observed, raising questions on the difficulty to carry out solution NMR studies on Bm-Aspin. Often, this kind of a situation specifies to the helical secondary structure of the protein under study and assuming that Bm-Aspin falls into that category, triple resonance NMR experiments were performed on the doubly labeled Bm-Aspin.

Thus in order to overcome the above difficulty, the following triple resonance NMR experiments, HNCO, HN(CO)CA, HNCA, CBCA(CO)NH and HNCACB were carried out at 298 K on a uniformly double ^13^C, ^15^N –labeled Bm-Aspin [45]. Protein sample was found to be stable throughout the data collection. From the analysis of the HNCO spectrum, 95% of the expected peaks with relatively uniform intensity were found. The severe overlap in the central region of the HSQC suggests a high helical content in Bm-Aspin. These findings are in agreement with the CD results that we published earlier suggesting the helical nature of Bm-Aspin [19]. Thus the three dimensional NMR experiments suggests the feasibility of conducting solution NMR-based structural studies on Bm-Aspin.

The peak overlaps in the HSQC spectrum of Bm-Aspin with a large number of amide peaks make its resonance assignment extremely challenging. The backbone assignment of Bm-Aspin was initiated by establishing the neighboring residue connectivity using pairs of triple resonance spectra, HNCA and HNCOCA; HNCACB and CBCA(CO)NH. The sequential NMR spin system connectivities have been achieved by both intra- and inter-residue cross-peaks of Cβ and Cα respectively. Any ambiguities were resolved by going through the ^15^N edited NOESY spectra. The usage of CBCA (CO) NH/HNCACB was found to be extremely useful not only for the improved resolution in the carbon dimension, but more importantly, it provided the phase information which will be used in the assignment process. The initial assignment was simple with the easier identification of Glycine residues and its connectivity with the other easily identifiable Ala, Ser and Thr residues. Nearly 15 residue-stretches with sequential residue connectivity have been identified and assigned. The complete backbone and side-chain assignments for the other residues of Bm-Aspin is in progress and will soon be communicated elsewhere.

### Bm-Aspin–Aspartic Protease interaction analysis using NMR

Before performing the NMR titration analysis between Bm-Aspin and human aspartic proteases, activity of pepsin in the above mentioned conditions (100 mM SDS and pH 7.0) was carried out. From these studies, it was clearly demonstrated that 100 mM SDS has no effect on pepsin activity and on the inhibitory activity of Bm-Aspin. The observation of protease digestion by pepsin at both pH 5.6 and 7.0 indicate a higher activity for pH 5.6, which is in agreement with earlier studies carried out on pH stability of pepsin [46]. In addition to this, 100 mM SDS seems to have negligible effect on pepsin activity. In fact, according to the literature, SDS was found to have least effect on the activity of many proteases. [47]–[51]. From these results, we felt that it's worth proceeding to study the aspartic protease binding property of Bm-Aspin using HSQC titrations.

The preliminary residue assignment of Bm-Aspin has been useful in the analysis of the Bm-Aspin interactions with the proteases. Our results clearly demonstrate that Bm-Aspin interacts with human aspartic proteases supporting our earlier results. The chemical shift perturbations were observed in the HSQC experiments upon the addition of aspartic proteases to Bm-Aspin. These observations provide the direct experimental proof that the Bm-Aspin contains the binding sites for human aspartic proteases. As expected, based on the displacement observed from the chemical shift perturbations in the HSQC of Bm-Aspin upon the addition of aspartic proteases, the inhibition slows down in the order of pepsin>renin>cathepsin-E>cathepsin-D. Similar findings were observed previously, when Bm-Aspin inhibition kinetic studies were carried out using UV-spectroscopy, suggesting the authentication of the experiments performed and the results obtained. However the detailed mechanism of protease inhibition by Bm-Aspin and its role in parasite survival can only be addressed further by structure-function studies with the determination of the solution structure of Bm-Aspin which would be initiated on completion of the assignment process.

### Conclusion

We report here the aspartic protease inhibition kinetic studies and feasibility to conduct the solution NMR based structural studies on Bm-Aspin. Aspartic protease inhibition assay carried out using the specific substrate suggested that Bm-Aspin inhibits the important human aspartic proteases supporting our earlier published data. Inhibition kinetic studies suggest that, Bm-Aspin follows competitive mode of inhibition for pepsin and cathepsin-E, non-competitive for renin, and mixed for cathepsin-D. The triple resonance NMR experiments conducted on Bm-Aspin with SDS detergent suggested the feasibility to carry out the NMR based structural studies. The activities of pepsin at pH 7.0 and in the presence of 100 mM SDS suggests that all the titrations carried out are in the biologically relevant form. The chemical shift perturbations were observed in the ^15^N HSQC spectra of Bm-Aspin upon the addition of aspartic proteases, suggesting the possibilities of identifying the binding sites of human aspartic proteases on Bm-Aspin. This work happens to be the first attempt on the solution NMR studies of the protein involved in human lymphatic filariasis. Considering the physiological role played by these human aspartic proteases, understanding the protease inhibition mechanism of Bm-Aspin will be interesting to speculate Bm-Aspin, as an important drug target for human lymphatic filariasis. For which, the high resolution 3D structure determinations of Bm-Aspin in apo-form by solution NMR and the complex with human pepsin by X-ray crystallography methods are well underway in our laboratories. These high resolution structures will be instrumental in our understanding of the structure/function and mechanism of aspartic protease inhibition.
